# Complex Polymeric Architectures Self-Assembling in Unimolecular Micelles: Preparation, Characterization and Drug Nanoencapsulation

**DOI:** 10.3390/pharmaceutics10040209

**Published:** 2018-11-01

**Authors:** Stefania Ordanini, Francesco Cellesi

**Affiliations:** Dipartimento di Chimica, Materiali ed Ingegneria Chimica “G. Natta”, Politecnico di Milano, Via Mancinelli 7, 20131 Milan, Italy; stefania.ordanini@polimi.it

**Keywords:** complex polymeric architectures, unimolecular micelles, unimolecular micelles characterization, drug delivery, nanoencapsulation

## Abstract

Unimolecular polymeric micelles are a class of single-molecule amphiphilic core-shell polymeric architectures, where the hydrophobic core is well stabilized by the hydrophilic shell, avoiding intermolecular core-core interactions. Multi-arm copolymers with a dendritic core, as well as hyperbranched and comb-like polymers, can form unimolecular micelles easily. In this review, examples of polymers able to form detectable unimolecular micelles will be presented, summarizing the analytical techniques used to characterize the unimolecular micelles and discriminate them from other supramolecular aggregates, such as multi-micelle aggregates. Unimolecular micelles are suitable for the nanoencapsulation of guest molecules. Compared to traditional supramolecular micelles, unimolecular micelles do not disassemble under dilution and are stable to environmental modifications. Recent examples of their application as drug delivery systems, endowed with increased stability and transport properties, will be discussed.

## 1. Introduction

**Figure 1:**
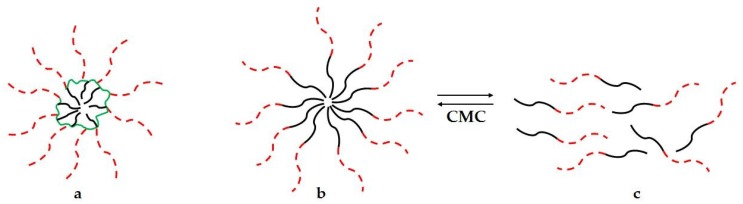
(**a**) Unimolecular micelles in aqueous solution are single-molecule architectures constituted by a hydrophobic core (black) and a hydrophilic shell (dashed red) covalently linked to a backbone (green); (**b**) conventional polymeric micelles in aqueous solution are an aggregation of amphiphiles having hydrophobic (black) and hydrophilic (dashed red) moieties; and (**c**) at a concentration below Critical Micelle Concentration (CMC), conventional micelles disassemble into free polymeric chains.

Unimolecular polymeric micelles are a class of single-molecule micelles having a core and a shell with two different polarities and covalently linked together [1,2,3]. Nano-sized unimolecular polymeric micelles are formed once appropriate amphiphilic polymeric molecules are dispersed in a selective solvent, i.e., a good solvent for the block with one polarity and precipitant for the block with the opposite polarity. In an aqueous solution, the hydrophobic polymeric core is efficiently wrapped and stabilized by the hydrophilic shell, avoiding core-core intermolecular interactions (Figure 1a) [4,5]. Differently from conventional polymeric micelles, which are thermodynamic aggregates of amphiphilic polymers above a certain concentration threshold, known as Critical Micelle Concentration (CMC) [6] (Figure 1b,c), unimolecular polymeric micelles are stable upon high dilutions, and could be formed below typically expected CMC values for the hydrophilic/lipophilic balance of the amphiphilic polymer [7].

Many polymeric amphiphilic species, having different topologies, can form unimolecular micelles. They are generally multi-arm polymers, where hydrophilic chains emanate from a central dendrimeric, hyperbranched or simply multivalent core [8]. It is known that increasing the complexity and the hindrance of polymeric structures, as well as the number of soluble branches, the formation of large micellar structures is less favored [9].

**Figure 2:**
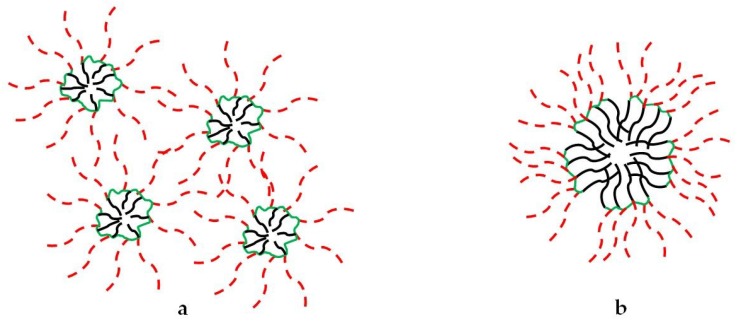
Unimolecular micelles can self-assemble forming (**a**) multi-micelle aggregates and/or (**b**) primary micelles.

The preparation of polymers able to form unimolecular micelles and their application as functional materials have recently been reviewed [1]; nevertheless, to the best of our knowledge, a systematic summary of the methods used to dissect their self-assembling behavior is missing. This would be beneficial to unambiguously assess the presence of unimolecular micelles or aggregates of unimolecular micelles, or the coexistence of more than one species. It is indeed possible that unimolecular micelles self-assemble in a kind of multi-micelle aggregates, characterized by an entanglement of the hydrophilic branches (Figure 2a), and/or they rearrange forming primary micelles, where the hydrophilic arms stabilize an aggregate of hydrophobic cores (Figure 2b) [10]. With this review, we aim to provide an overview of the techniques that have been employed to detect and characterize unimolecular micelles.

One of the main advantages of unimolecular micelles compared to the conventional ones concerns their application as drug delivery systems [11,12,13]. Indeed, amphiphilic polymers can self-assemble in aqueous solutions accommodating guest molecules within their core. This ability is exploited to encapsulate hydrophobic drugs, thus improving their solubility and their circulation time in the blood, protecting them from the surrounding environment and non-specific uptake by the mononuclear phagocyte system [14]. Conventional micelles subjected to high dilutions in the bloodstream might disassemble [15], leading to burst release of the encapsulated drug, possible toxic effects related to high variations of local drug concentration, and finally the inability to reach the targeted site. In this review, recent examples of thermodynamically stable unimolecular micelles used to transport poorly soluble hydrophobic drugs to targeted organs/tissues will also be presented.

## 2. Preparation of Unimolecular Micelles

In order to prepare unimolecular polymeric micelles in aqueous solution, a dense hydrophilic shell, able to stabilize the core, is needed. As a matter of example, highly branched macromolecules, including multi-arm and hyperbranched polymers, are commonly stabile and non-entangling [4]. Tailoring the structure of polymeric molecules is therefore essential. Moreover, polymers compositions and final molecular weights affect the size of resulting micellar structures, and therefore the scope of their applications. In recent reviews, various types of amphiphilic polymers with complex architectures that have been applied in the fabrication of unimolecular micelles have been reported [1,16]. Amphiphilic dendrimers, hyperbranched polymers, star polymers, other types of amphiphilic polymers (bottlebrush [17,18], Y shaped [19,20], and cyclic [21,22]) have been designed for this purpose. Controlled polymerization techniques (such as Ring Opening Polymerization (ROP) and Atom Transfer Radical Polymerization (ATRP)), which allow a good control over the structure, the molecular weight and the polydispersity of the synthesized macromolecules [23], are generally exploited, often in combination with “grafting through”, “grafting onto”, or “grafting from” approaches to obtain amphiphilic graft copolymers [1]. Examples of multi-arm and hyperbranched polymers, characterized by a complex and controlled architecture and able to form detectable and able to be characterized unimolecular micelles, will be presented hereafter.

### 2.1. Multi-Arm Polymers

Unimolecular micelles can be obtained from a variety of different multi-arm polymers. Multi-arm star amphiphilic block copolymers built on hydrophobic H40, a fourth generation dendrimeric polyester (Boltorn-type), have been reported [24,25,26]. Polymers can be synthesized either initiating the ROP of lactones with H40-OH and finally coupling the resulting polyesters with hydrophilic poly(ethylene glycol) (PEG) chains, or coupling amphiphilic polyester-PEG chains onto H40 via ester linkage.

**Figure 3:**
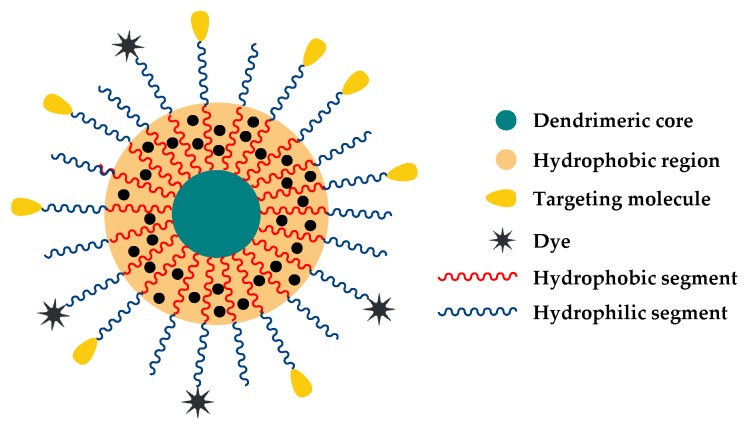
Schematic representation of the unimolecular micelle formed by multi-arm star amphiphilic block copolymer PAMAM-PVL-PEG-OCH_3_/Cy5/KE108. Reproduced with permission from reference [28], Elsevier, 2016.

Multi-arm polymers with an aromatic dendritic polyphenylene core were reported by Yin et al. [27]. The shell of poly(2-aminoethyl) repeating units was obtained by ATRP of *N*-2-[(tert-butoxycarbonyl)amino] ethyl methacrylate (Boc-AEMA), followed by Boc deprotection. Gong and coworkers built a multi-arm star amphiphilic block copolymer starting from a polyamidoamine (PAMAM) dendrimeric core [28]. PAMAM-OH (generation four) served as the macroinitiator for the ROP of valerolactone. The resulting hydrophobic segments were then activated and pegylated. Final polymer PAMAM-PVL-PEG-KE108/Cy5, able to form unimolecular micelles in water (Figure 3), was obtained after a further functionalization with a targeting peptide (KE108) and a dye (Cy5).

**Figure 4:**
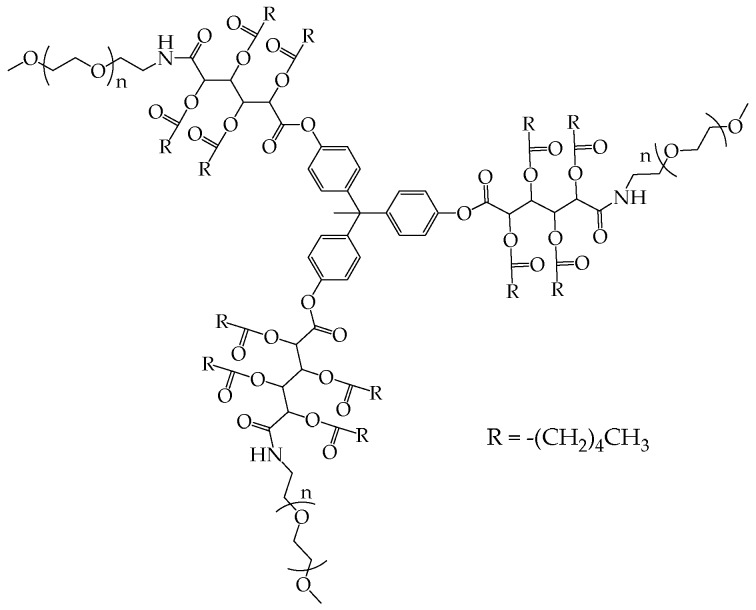
Multi-arm polymers based on a poly-alkylated hydrophobic core and a pegylated hydrophilic shell. Reproduced with permission from reference [29], Royal Society of Chemistry, 2000.

A trivalent aromatic scaffold, 1,1,1-tris(4’-hydroxyphenyl)ethane, was used by the group of Uhric to synthesize a multi-arm polymer with a pegylated hydrophilic shell and an inner hydrophobic core constituted by mucic acid functionalized with several fatty acids (e.g., hexanoyl chloride, Figure 4) [29].

**Figure 5:**
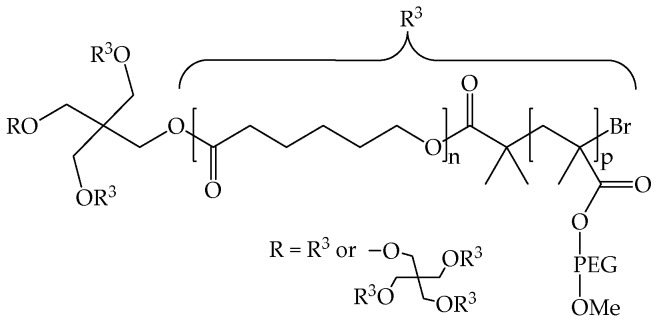
Amphiphilic multi-arm star-shaped block copolymers based on a poly(ε-caprolactone) core and a pegylated comb-like shell. Reproduced with permission from reference [30], Royal Society of Chemistry, 2009.

The group of Schubert reported the formation of unimolecular micelles from amphiphilic multi-arm comb-like polymers. Pentaerythritol or dipentaerythritol initiated the ROP of ε-caprolactone (CL); resulting polyester chains were then converted into 2-bromoisobutyryl derivatives and used as macroinitiators of the ATRP of poly(ethylene glycol) methyl ether methacrylate (PEGMA). By varying the number of CL and PEGMA repeating units, a library of polymers with the general structure described in Figure 5 was obtained [30]. In another study, these polymer architectures were used to generate steroid-loaded ultrasmall nanocarriers for targeted delivery to the kidney glomerulus [31].

### 2.2. Hyperbranched Polymers

Hyperbranched polymers have been widely used for unimolecular micelle preparation. In contrast to the multi-step synthesis of precisely defined dendrimers, hyperbranched macromolecules can be synthesized in much easier approaches, which are often based on convenient one-step syntheses [1].

**Figure 6:**
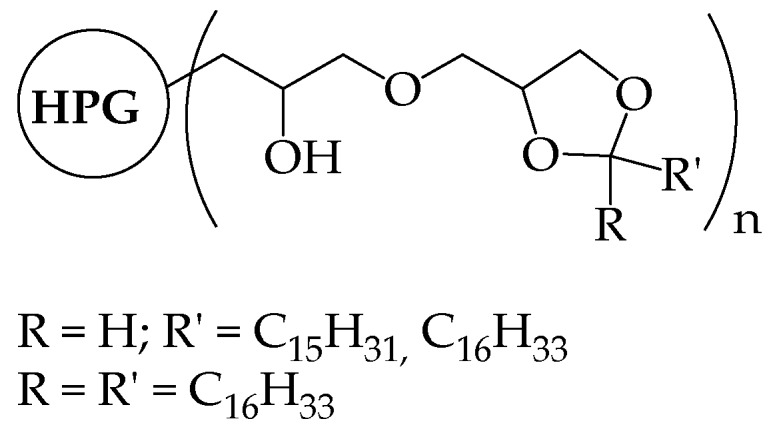
A hydrophilic hyperbranched polyglycerol, whose vicinal hydroxyl groups were partially functionalized as acetals or ketals by reacting with aldehydes or ketons with long alkyl chains. Reproduced with permission from reference [32], Wiley, 2002.

Amphiphilic hyperbranched polymers can be synthesized by functionalizing polyglycerol. The group of Haag generated unimolecular micelles (R_h_ ≤ 5 nm, AFM) by selectively functionalizing the terminal hydroxyl groups of hyperbranched polyglycerol (M_n_ = 21,000 Da) with long alkyl chains (C_15_, C_16_). pH-responsive nanocarriers, able to encapsulate polar guest molecules, were obtained by using acetals/ketals acid-sensitive linkages (Figure 6). The shell cleavage under acidic conditions resulted in cargo release [32]. By using a similar synthetic approach, reverse unimolecular micelles were also obtained in cyclohexane from hyperbranched polyglycerols, whose hydroxyl groups were partially functionalized with palmitoyl chloride [33].

**Figure 7:**
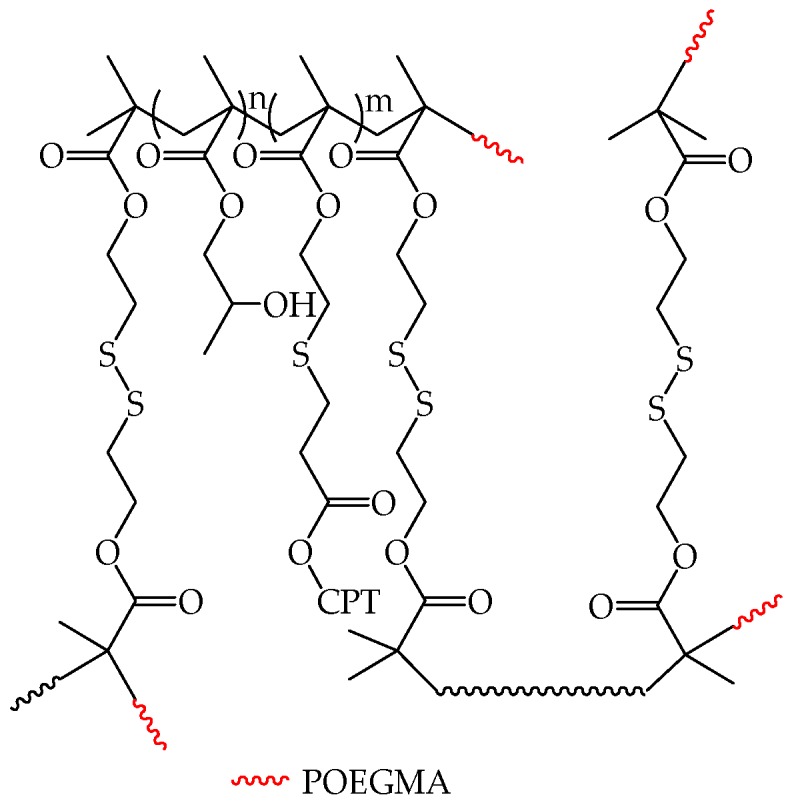
Hyperbranched amphiphilic star copolymers with a HPMA-*co*-MACPT-*co*-BS_2_MOE core and a POEGMA (red) hydrophilic shell. Reproduced with permission from reference [34], Royal Society of Chemistry, 2016.

Qui et al. obtained pH- and redox-responsive unimolecular micelles from camptothecin (CPT)-conjugated hyperbranched star copolymers containing acid-labile β-thiopropionate linkage [34]. These HP(HPMA-*co*-MACPT-*co*-BS_2_MOE)(POEGMA)_n_ copolymers (Figure 7) were characterized by hyperbranched macroinitiators functionalized with hydrophilic poly-oligo(ethylene glycol) (POEGMA). Macroinitiators were obtained by ATRP of hydroxypropyl methacrylate (HPMA), methacryloyloxy-3-thiohexanoyl-camptothecin (MACPT), and 2-(2′-bromoisobutyryloxy)ethyl-2″-methacryloyl oxyethyl disulfide (BS_2_MOE), which acted as the inimer (i.e., the branching agent being simultaneously the initiator and the propagating species) [34]. The obtained polymers formed unimolecular micelles in water, and their release of CPT at various pHs as well as their anticancer efficacy was confirmed in vitro.

Amphiphilic hyperbranched fluoropolymers were proposed as nanoscopic ^19^F Magnetic Resonance Imaging Agents [35]. Atom transfer radical self-condensing vinyl (*co*)polymerization (ATR-SCVCP) of 4-chloromethyl styrene in the presence of lauryl acrylate, initiated by 1,1,1-tris(4′-(2′′-bromoisobutyryloxy)phenyl)ethane (TBBPE), yielded the hydrophobic core of a hyperbrached polymer, finally obtained by subsequent polymerization of trifluoroethyl methacrylate (TFEMA) and *tert*-butylacrylate. The fluorinated amphiphilic polymers were obtained after restoring the acidic functionality of the acrylic acid, and they formed unimolecular micelles in water [35].

## 3. Characterization Methods

### 3.1. Characterization of Unimolecular Micelles

One of the most common methods used to assess the presence of unimolecular micelles in solution is used to compare their size with the size of the coils of a single polymeric molecule in solution (predicted or experimentally determined). The size of unimolecular micelles is often determined by means of Dynamic Light Scattering (DLS) analysis [36]. For instance, the hydrodynamic diameter D_h_ of micelles formed by HP(HPMA-*co*-MACPT-*co*-BS_2_MOE)(POEGMA)_n_ polymers (Figure 7) [34] in water varied from 3.5 to 6.3 nm, increasing as a function of polymers molecular weight. D_h_ values were in line with the size of isolated polymeric molecules, estimated with the length of chemical bonds.

Haag et al. predicted the size of a hyperbranched polyglycerol with M_n_ = 21,000 Da (Figure 6) according to force-field calculations and mass-volume correlations [32].

In several examples, the average size values measured by DLS are compared with data obtained by Transmission Electron Microscopy (TEM) [24,25,35,37,38]. The DLS analyses of PAMAM-PVL-PEG-KE108/Cy5 micelles [28] showed a mean D_h_ of 56 nm, in line with the size determined by TEM microscopy (D_h_ = 38 nm), which disclosed spherical nanoparticles with a clear core-shell structure.

Generally, diameters measured by DLS are slightly higher than those measured by TEM, because in the first case micelles are in an aqueous solution, where hydrophilic components swell and extend, whilst in the second case, they are dried [24,36].

In case of bottlebrush micelles, small-angle X-ray and neutron scattering (SAXS, SANS) can also be used to examine micelle morphology and internal structure, and to obtain information regarding the overall size of the polymer and cross-sectional dimensions, thus evaluating the intermolecular assembly and possible formation of large aggregates [39,40].

In many reports micelle size in water or another selective solvent, obtained by DLS, is compared with the size that they have in a non-selective solvent, where they are soluble, isolated and not prone to form aggregates. As an example, the size in aqueous buffer (selective solvent) of poly(phenylene)-star-poly(aminoethyl methacrylate) was comparable with the one of their Boc-precursors in dichloromethane (non-selective solvent) [27]. Moreover, the value of hydrodynamic radius calculated at different scattering angles did not change, and this corroborated the existence of isolated and well-defined species [41]. Star-shaped PCLp(mPEGMA)s, depicted in Figure 5, had a similar size in water or THF, slightly higher in water, likely because of the PEGMA shell swelling [10].

In contrast to conventional micelles, unimolecular polymeric micelles do not form according to a CMC transition. There are several experimental methods that can be used to assess this behavior. Uhric et al. showed that the surface tension profile of the polymers in Figure 4, dissolved in aqueous solution at a concentration ranging from 10^−4^ to 10^−6^ M (corresponding to ~2–0.02 mg/mL), did not have a distinct change of slope [29]. The lack of CMC was considered as the proof of the lack of intermolecular polymeric aggregations. Following an analogous procedure, concentration dependent DLS analyses were performed on PCLp(mPEGMA)s comb-like polymers (Figure 5) [10]. As expected, the derived count rate increased as a function of concentration, but never showed a sudden change, which would have been attributed to a transition from unimolecular micelles to multi-micelle aggregates. Plotting the number of scattered photons determined by light scattering analysis vs. concentration is another way to calculate CMC values [42]. The constancy of the hydrodynamic radius itself with respect to concentration can corroborate the lack of aggregating behaviors [33].

Fluorescent probes can also be used to determine critical micelle concentration values; pyrene is probably the most exploited one. It is an aromatic dye having a low water solubility (<1 mg/L) and a different fluorescent behavior according to the polarity of the medium [43]. In the presence of amphiphiles, a rapid change of the ratio of the pyrene emission bands I_370_ to III_390_ indicates the transition from single molecules to micelles, able to solubilize the dye in their hydrophobic core [44]. It could also indicate a transition from unimolecular micelles to multi-micelle aggregates, if the first ones are unable to encapsulate pyrene molecules. Oil Red O or Nile Red can also be used for this purpose; a fluorescent emission signal is detected only when they are solubilized in the medium, namely, they are encapsulated in a hydrophobic domain. It has been observed that some amphiphilic polymeric species showing a CMC with the pyrene assay lacked a CMC when using Oil Red O or Nile Red dyes [10,45]. Schubert and coworkers claimed that pyrene assay is not always reliable, since the change of the ratio of band I to band III can be affected by the dye excimer formation if the dye concentration exceeds the polymer encapsulation capacity [10]. At the same time, a transition from unimolecular to multimolecular micelles might not be detected by those dyes that can be solubilized by both of the species. Since the dyes activity varies according to their polarity and their affinity for the micellar cores, which might be difficult to be predicted a priori, a comparison of different analytical techniques would be recommended to get a more reliable picture of the micelles real morphological behavior.

### 3.2. Characterization of Multi-Micelle Aggregates

The formation of unimolecular micelles and their coexistence with larger associated micelles has been reported in several papers [38,46].

Hong et al. synthesized multi-arm polymers with a hyperbranched poly(3-ethyl-3-(hydroxymethyl)oxetane) (HBPO) core and a shell constituted by many poly(2-(dimethylamino)ethyl methacrylate) (PDMAEMA) chains [47]. The presence of two distinct species in water, having a D_h_ of about 10 and 100 nm, respectively, was assessed by both DLS and TEM analyses. The smallest species were considered the unimolecular micelles, since their sizes were comparable with the length of the PDMAEMA chain (i.e., 18 nm, if it adopted a completely zig–zag stretched conformation). As clearly shown by TEM images, the hundred-nanometer-sized species were multi-micelle aggregates built up from the aggregation of unimolecular micelles. The coexistence of the two species was observed at 10 mg/mL, far above their CMC, which was 0.02–0.05 mg/mL (measured with both the pyrene and the Nile Red dyes). Below this concentration value, only unimolecular micelles likely exist.

The group of Haag reported a multi-shell complex polymer constituted by a hydrophilic dendritic poly(ethylene imine) (PEI) core, a nonpolar inner shell formed by alkyl diacids (C_6_, C_12_, or C_18_) and, finally, a polar pegylated outer shell [48]. The D_h_ of unimolecular molecules in water was predicted to be approximately 6 nm by molecular modeling, and a CMC of 0.1 mg/mL was calculated by surface tension analysis. Additionally, in this case, DLS and TEM analyses in water, performed at a concentration of 0.5–10 mg/mL and 1 mg/mL, respectively, showed the coexistence of unimers (D_h_ ~ 5 nm) and aggregates (D_h_ ~ 30–50 nm) formed by a large number of unimolecular species. Authors attributed the self-assembly process to the aliphatic chains, and the additional stabilization of the aggregates to the polyethylene glycol chains.

Similarly, a multi-arm polymer HCP-N-PEG, synthesized by pegylating an hyperbranched conjugated polymer (HCP) based on tri-substituted benzene and *N*-alkylated carbazole moieties, yielded unimolecular and multimolecular micelles in water [49,50]. The two species were simultaneously detected by means of DLS and TEM analyses. Interestingly, the core molecules are fluorescent, and a new emission peak appears when they collapse together. The lack of this new peak in the fluorescent spectrum of HCP-N-PEG micelles in aqueous solution demonstrated that big aggregates are formed by the self-assembly of unimolecular micelles and not by intermolecular aggregation of HCP cores [51].

A multi-arm amphiphilic block copolymer, based on a PAMAM-OH (generation 2) core, functionalized with poly(ε-caprolactone)-polyethylene glycol (PCL-PEG) copolymers, formed both unimolecular and aggregated micelles in water, with a CMC of 3 mg/mL (pyrene test) [45]. The two species, with a D_h_ of 16.9 nm and a 75 nm, respectively, according to DLS analyses (12 mg/mL), were detected also by size exclusion chromatography in aqueous solution. Aggregates might result from hydrophobic-hydrophobic interactions or van der Waals interactions between the exposed hydrophobic PCL cores of the single polymeric molecules. Remarkably, no CMC value was detected using Oil Red O (ORO) as fluorescent dye; this was probably due to the fact that ORO, being more polar than pyrene, has a higher compatibility with the core of unimolecular micelles, which is expected to be less hydrophobic than the one of micelle aggregates.

## 4. Unimolecular Micelles as Drug Delivery Systems

As complex polymeric architectures may self-assemble forming stable unimolecular micelles, they can encapsulate and transport poorly soluble hydrophobic drugs, enhancing the therapeutic efficacy and reducing the side effects in vivo [52].

Nowadays, most of the nanocarrier systems used in nanomedicine are designed for anticancer therapies, which often relies on the so-called Enhanced Permeability and Retention effect (EPR) to passively target and reach tumors [53]. On the other hand, active targeting can be performed by functionalizing nanoparticles surface with molecules (e.g., peptides, antibodies) specifically recognized by tumor or other targeted cells [54].

In this section, recent examples of unimolecular micelles for drug delivery will be presented, especially highlighting the in vivo applications.

### 4.1. Drug Loading and Release

The drug loading is generally achieved by solvent evaporation or dialysis methods, as developed for standard polymer micellar systems [55,56]. Typically, the drug and the polymer are dissolved in an organic solvent where the two compounds are both miscible [57]. Water or buffer is added directly to the solution to cause micelle formation, then the organic solvent (e.g., acetone, THF, acetonitrile) is removed by nitrogen flux or vacuum evaporation, and the encapsulation of the drug is finally obtained. When the solvent is not easily removable by evaporation (such as DMF, DMSO), a dialysis can be used. Alternatively, the organic polymer-drug solution is dried and the resulting matrix is hydrated afterwards by H_2_O or buffers (PBS) to obtain drug-loaded micelles [58]. The characterization of drug-loaded micelles is similar to other nanoparticles. Parameters of interest are the drug loading (DL) and the drug encapsulation efficiency (EE), which are defined as DL = W_L_/W_N_ and EE = W_L_/W_0_, where W_L_ is the weight of loaded drug, W_N_ the weight of the nanoparticle, and W_0_ the quantity of drug initially added in the formulation. The typical drug loading DL is in the range 1–20 wt% for unconjugated drugs.

The in vitro release studies are generally carried out by transferring the drug-loaded micelles into dialysis membrane tubing (at a molecular weight cut-off below the polymer average molecular weight) in containers filled with buffers which are maintained at physiological pH and temperature [50,59]. The tests are carried out under sink conditions [15,50], i.e., the total volume of the release medium is chosen so that when drug is completely released the concentration is below its solubility [59]. The amount of drug released at different time-points is usually quantified by HPLC or fluorescence. In general, the release profiles are characterized by a rapid-release phase followed by a slower sustained-release phase, and they are affected by the drug-polymer interactions and by the drug solubility in the medium [15,50,59].

### 4.2. Biocompatibility

In drug delivery, it is crucial to evaluate the factors that affect the biocompatibility of the nanocarriers, in order to ensure safe drug release. Biocompatibility of unimolecular micelles is generally achieved when they interacts with the host without inducing unacceptable toxic, immunogenic, thrombogenic, and carcinogenic responses, as in the case of any type of polymeric nanoparticles [60]. These responses are typically dependent on polymer composition and biodegradability, particle size, and surface properties [61]. Complex polymeric architectures based on a biodegradable polyester and PEG blocks usually guarantee low cytotoxicity, minimize long term in vivo accumulation, and limited activation of the mononuclear phagocyte system (MPS). In fact, polymers such as PLA, PCL, and PEG are considered adequately biocompatible by the regulatory agencies such as the US Food and Drug Administration (FDA) and the European Medicines Agency (EMEA) [60,61].

However, metal catalysts are commonly used in controlled polymerizations such as ROP and ATRP, and some studies have pointed out their toxicity for human health, as they tend to accumulate in the body, where they interact with enzymes and other biomolecules, and are involved with redox reactions [62,63]. Since the removal of metal impurities from synthetic polymers is generally expensive, organocatalysts have been used for the preparation of FDA-approved polyesters [62]. “Green” ATRP strategies have also been proposed as an alternative to traditional ATRP, which needs a relatively large amount of copper catalyst (typically of the order of 0.1–1 mol% relative to monomer) [63]. For instance, the copper amount can be highly reduced by using activators generated by electron transfer (AGET) ATRP [62]. Alternatively, it can be substituted by a more biologically friendly iron catalyst [64].

While polyesters are hydrolyzable in a biological environment, the unimolecular micelles may also contain non-biodegradable polymer sequences (PEG blocks, vinyl (carbon-carbon) backbones, non-degradable multifunctional cores). Therefore, the complex macromolecules should be metabolized/degraded to yield low-molecular-weight compounds or macromolecular products with molecular weight lower than 10–50 kDa, which can be finally excreted by the kidneys [65,66]. Non-degradable polymers that are too large to undergo renal clearance may be retained in the body for an extended period of time, with possible long-term nanotoxic effects [67].

Due to the high complexity of the chemical and physical phenomena, which are involved in the nanocarrier-host interactions, the biocompatibility of unimolecular micelles should be tested on a case-by-case basis. Cytotoxicity tests (such as MTT assay or similar), as well as the evaluation of the effect of the nanomaterial on intracellular reactive oxygen species (ROS) levels and/or the levels of pro-inflammatory mediators, may be carried out in vitro [68]. Nanomaterial biodistribution and clearance, which are mainly affected by opsonization and MPS activation/inhibition, need to be carried out in vivo [69].

### 4.3. Multi-Arm Copolymers for Drug Delivery

Several studies reported the use of multi-arm star block copolymers for the encapsulation and release of chemotherapeutic agents in mice and rats. One kind of multi-arm topology is achieved by generating the copolymer from the H40 (Boltorn-type) dendrimeric polyester [70,71,72,73,74]. The ring-opening polymerization of ɛ-caprolactone (CL) from the periphery of H40 and subsequent ATRP of oligo(ethylene glycol) monomethyl ether methacrylate (OEGMA) and 3-azidopropyl methacrylate (AzPMA) was used to obtain well-defined multi-arm star block copolymers, H40-PCL-b-P(OEGMA-*co*-AzPMA), which were conjugated by the click reaction with the alkynyl-functionalized cancer cell-targeting moiety alkynyl-folate, and a T1-type MRI contrast agent, alkynyl-DOTA–Gd (DOTA is 1,4,7,10-tetraazacyclododecane-1,4,7,10-tetrakisacetic acid), affording H40-PCL-b-P(OEGMA-Gd-FA) [70]. This polymer was capable of encapsulating paclitaxel and provided positive contrast enhancement in MR imaging, revealing good nanocarrier accumulation within rat liver and kidney, and relatively long blood circulation.

Different studies reported the use of acid-cleavable (due to the presence of acetal groups) H40-PCL-PEG based micelles for intracellular drug release and enhanced cancer therapy [75], since the tumor microenvironment usually has lower pH compared to normal tissues and blood [76]. Enhanced intracellular drug release has also been obtained by H40-PCL-based redox responsive polymers, which presented a redox-sensitive (disulfide) cross-linker for glutathione-mediated intracellular drug delivery [77].

In another study, H40 was used to generate amphiphilic polylactide-poly(ethylene glycol) (PLA-PEG) block copolymer arms. In order to achieve active neuroendocrine (NE) tumor targeting, octreotide (OCT), a somatostatin analog, with a strong binding affinity to somatostatin receptors (SSTRs), was conjugated to the PEG outer shell, to obtain the final polymer H40-PLA-PEG-OCH_3_/OCT [71]. The resulting micelles were loaded with the histone deacetylase inhibitor thailandepsin-A (TDP-A). Tests on NE cancer-bearing nude mice demonstrated that these nanomaterials provided superior anticancer activity in comparison with other TDP-A formulations, and showed a significantly reduced systemic toxicity.

A similar H40-PLA-PEG polymer was functionalized with an aptamer (A10) that can specifically recognize the extracellular domain of the prostate-specific membrane antigen (PSMA), abundantly expressed on the surface of the prostate cancer (PCa) cells [73]. Doxorubicin (DOX) was physically encapsulated into the hydrophobic core of these unimolecular micelles and the H40-PLA-PEG-Apt exhibited a much higher level of DOX accumulation in the tumour tissue of tumour-bearing mice, with respect to the non-targeted DOX-loaded H40-PLA-PEG micelles.

A dendritic amphiphilic block copolymer H40-poly(d,l-lactide)-block-d-α-tocopheryl polyethylene glycol 1000 succinate (H40-PLA-b-TPGS) was synthesized and loaded with docetaxel (DTX) [72]. The antitumor effect of this system was confirmed in mice by xenograft tumour model with intraperitoneally injected human breast carcinoma MCF-7 cells, and it was significantly superior to those of the linear copolymers and the free drug.

β-cyclodextrin (CD) was also used as multivalent scaffold to build multi-arm block copolymers of poly-lactic acid (PLA), poly-2-(dimethylamino)ethyl methacrylate (PDMAEMA) and poly-oligo(2-ethyl-2-oxazoline)methacrylate (PEtOxMA) [78]. PDMAEMA, containing tertiary amines, made the polymers pH responsive. As predicted by Particle Dynamic Simulations (DPD), block copolymers formed unimolecular micelles in water solution, with an average size of 19 nm (DLS), and no evidence of CMC (Nile Red assay). Unimolecular micelles were used to encapsulate imiquimod (IMQ), an immunostimulatory hydrophobic drug (DL = 1.6 wt%); as expected, the drug release was faster at acidic pH. Additionally, micelles were used to condense plasmid DNA through the cationic amino groups of the PDMAEMA chains. Micelliplexes with the average diameter ranging from 150 to 400 nm, were efficiently formed and preliminarily tested in a mouse dendritic cell line as dual functional drug and gene nanocarriers.

β-cyclodextrin was used as initiator to generate the 21-arm star-like triblock polymer β-cyclodextrin-{poly(ε-caprolactone)-poly(2-aminoethyl methacrylate)-poly[poly(ethylene glycol) methyl ether methacrylate]}_21_ [β-CD-(PCL-PAEMA-PPEGMA)_21_], by combining ROP with ATRP techniques [79]. The resulting unimolecular micelles formed in aqueous solution were used as a template for fabricating gold nanoparticles (AuNPs) with uniform size and for the encapsulation of doxorubicin (DOX). Tests on mice bearing human hepatocellular carcinoma HepG2 tumours demonstrated that these theranostic nanomaterials exhibited similar efficacy to free DOX and effective CT imaging performance.

**Figure 8:**
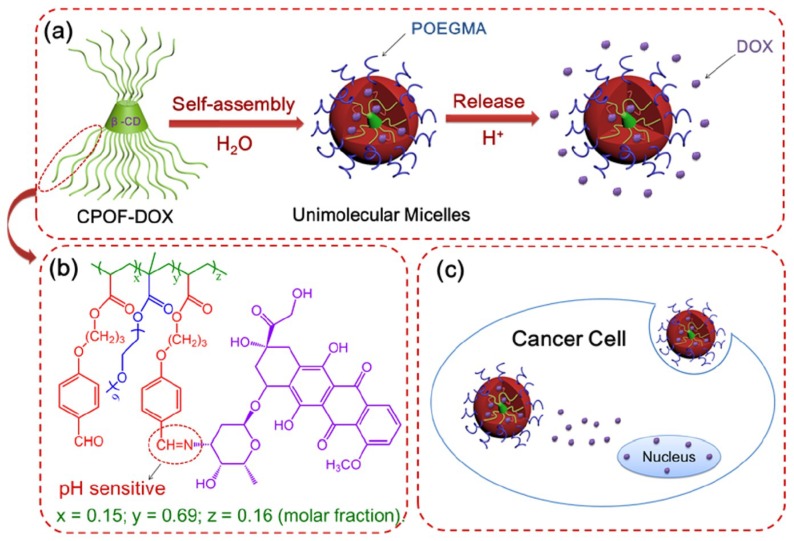
(**a**) Schematic representation of β-cyclodextrin-based copolymers, forming unimolecular micelles in aqueous solution. In acidic media, they release doxorubicin (purple dots); (**b**) Chemical structure of each arm of the multi-arm polymer. Doxorubicin is connected to the benzaldehyde-bearing monomer via a Shiff-base linkage; and (**c**) unimolecular micelles are internalized by HeLa cells, and after being released, doxorubicin accumulates in the nuclei. Reproduced with permission from American Chemical Society. Reproduced with permission from reference [81], American Chemical Society, 2017.

Rather than encapsulating the therapeutic molecule within the core of the micelles, another drug delivery approach consists of incorporating the drug into the micelle via a covalent bond. Doxorubicin was conjugated to β-cyclodextrin (CD)-based multi-arm amphiphilic random copolymers [80], which consisted of hydrophilic poly-oligo(ethylene glycol) methyl ether methacrylate and 3-(4-formylphenoxy) propyl acrylate repeating units; the last ones were further functionalized with DOX, forming an acid-labile Schiff–base linkage (Figure 8) [81]. Polymers in aqueous solution formed nanoparticles with D_h_ = 16 nm (DLS); the size did not change when the concentration was lowered from 500 to 50 μg/mL, thus indicating the presence of stable unimolecular micelles (Figure 8a). Micelles released the 20% of DOX over 50 h at pH 7.2, and the 50% at pH 5.0; this sustained release would be beneficial to avoid undesired drug leaching and toxicity toward healthy tissues. Micelles were internalized by HeLa cells and finally localized in the cytoplasm in the first 12 h. By increasing the incubation time to 24 h, DOX was released by polymers and accumulated within the nuclei (Figure 8c).

A similar approach involved the use of Boltorn H40 rather than β-CD as macromolecular core. H40-poly(l-glutamate-hydrazone-doxorubicin)-b-poly(ethylene glycol) (H40-P(LG-Hyd-DOX)-b-PEG) was conjugated with cRGD peptide (for integrin targeting) and macrocyclic chelators (NOTA) for ^64^Cu-labeling and PET imaging [74]. DOX was covalently conjugated via hydrazone linkage onto the hydrophobic segments of the amphiphilic block copolymer arms (i.e., PLG) to enable pH-controlled drug release. In tumor-bearing mice, H40-DOX-cRGD-^64^Cu also exhibited a much higher level of tumor accumulation than H40-DOX-^64^Cu, as measured by non-invasive PET imaging and confirmed by biodistribution studies.

Positively charged carriers, such as polyamidoamine (PAMAM) dendrimers, have a concentration-dependent cytotoxicity [82] and may non-specifically interact with biomolecules, possibly resulting in aggregated structures that are easily recognized by the immune system [83]. Using PAMAM dendrimers as scaffolds in more complex architectures, for example to generate multi-arm block copolymers, can be an alternative strategy for generating unimolecular micelles, exploiting their ability to transport drugs, while weakening their side effects.

Generation 4 PAMAM (PAMAM-G4) was used as macroinitiator for the formation of PLA arms followed by PEG conjugation and final grafting with the GE11 peptide, which targets epidermal growth factor receptor (EGFR), i.e., a protein which is frequently overexpressed in triple negative breast cancer (TNBC) tumors [84]. The polymer was also conjugated with the Cy5.5 fluorophore and the resulting PAMAM-PLA-PEG-OCH_3_/Cy5.5/GE11 unimolecular micelles were used to encapsulate aminoflavone (AF), a drug which exhibits strong growth inhibitory effects in TNBC cells. The nanoformulation significantly inhibited orthotopic TNBC tumour growth in a xenograft mice model, compared to treatments with AF-loaded, GE11-lacking (non-targeted) unimolecular micelles or free AF.

PAMAM dendrimer was also used to generate amphiphilic poly(δ-valerolactone)-poly(ethylene glycol) (PVL-PEG) block copolymer arms [85]. They were further conjugated with KE108 peptide or octreotide (OCT), i.e., somatostatin analogs that display strong binding affinities to medullary thyroid cancer (MTC) cells, and Cy5 dye. The histone deacetylase inhibitor AB3, which can effectively inhibit MTC cell proliferation, was successfully encapsulated by these polymers. These AB3-loaded, KE108-conjugated micelles demonstrated the best anticancer efficacy in mice without any apparent systemic toxicity. The same polymer system (PAMAM–PVL–PEG–KE108/Cy5) was used for the encapsulation of thailandepsin-A (TDP-A), a recently discovered histone deacetylase (HDAC) inhibitor [28]. In neuroendocrine (NE)-tumour-bearing nude mice, this nanoformulation showed enhanced tumour accumulation due to its passive and active targeting capabilities and evident anticancer efficacy without detectable systemic toxicity.

An analogous PAMAM (generation 4) derivative, connected to a PVL-PEG copolymer and functionalized with the cholera toxin B domain (CTB), was used to target and protect retinal ganglion cells (RGC), commonly lost in the glaucoma disease [86]. Unimolecular micelles with an average D_h_ of 62 nm in aqueous solution were loaded with the neurosteroid dehydroepiandrosterone (DHEA, DL = 18.6 wt%), which is known to have a protective role on retinal neurons. The drug was quickly released under acidic conditions (pH 5.3), probably because of the fast degradation of the polyester shell; a sustained release for at least two months was observed, which is a critical parameter to increase long-term drug efficacy against chronic pathologies. In vivo assays based on intravitreal injection of loaded unimolecular micelles were carried out in mice with induced RCG degeneration. Targeted micelles accumulated at the mouse RCG layer, contrarily to non-targeted ones. The therapeutic effect of DHEA-targeted unimolecular micelles in preserving RCG cells was supported by their stability in vivo, especially considering the high dilution and clearance in the eye environment.

PAMAM-PVL-PEG unimolecular micelles were also embedded in a synthetic biocompatible hydrogel to encapsulate rapamycin and obtain prolonged steady drug release [87]. The perivascular application of this hybrid system in rats produced a pronounced inhibitory effect on intimal hyperplasia, thus exhibiting a real potential for mitigating restenosis after open surgery.

Biodegradable dendritic block copolymer poly(amidoamine)-polyglutamic acid-b-poly(ethylene glycol) (PAM-PGlu-b-PEG), which was obtained from generation 3 PAMAM (PAMAM-G3) as a core, was used to encapsulate and release the antitumour agent 1,2-diaminocyclohexane-platinum(II) (DACHPt) for enhanced therapy of lung cancer [88]. The system demonstrated efficient antitumor efficacy in mice on a xenograft tumour model with negligible tissue cytotoxicity.

Nanocarriers bearing fluorescent moieties act as theranostic devices, being both imaging and drug delivery systems. A library of multi-arm polymers based on a π-conjugated poly(fluorene-alt-(4,7-bis(hexylthien)-2,1,3-benzothiadiazole)) (PFTB) backbone, with intrinsic strong FR/NIR fluorescence, formed stable and fluorescent unimolecular micelles in aqueous solution [89]. PFTB was connected to linear poly(ε-caprolactone) and comb-like poly-oligo(ethylene glycol) methyl ether methacrylate chains, yielding PFTB-g-(PCL-b-POEGMA) copolymers. Their DOX-loaded unimolecular micelles were internalized by both L929 and HeLa cells, and their high fluorescence allowed their detection in the cytoplasmic and perinuclear regions.

The fluorescent perylene-3,4,9,10-tetracarboxylic acid diimide (perylene diimide, PDI) was used as initiator to generate a novel PDI-core star block copolymer poly(d,l-lactide)-b-poly(ethyl ethylene phosphate), named as PDI-star-(PLA-b-PEEP)_8_ [90]. The resulting unimolecular micelles self-assembled into fluorescent supramolecular micelles (FSMs) with controllable sizes in aqueous solution, and the potent hydrophobic anticancer drug camptothecin (CPT) was chosen as model drug for nanoencapsulation. Tumour growth-inhibitory studies in MDA-MB-231 tumor-bearing mice revealed a better therapeutic effect of FSMs after CPT encapsulation when compared with free CPT.

Redox-responsive star-shaped micelles were obtained from four-arm poly(ε-caprolactone)-poly(ethylene glycol) copolymer [91]. The star-PCL polymer was synthesized by ROP of ε-caprolactone (ε-CL) initiated by pentaerythritol. The redox-responsive behaviour was realized by connecting the PCL and PEG segments with disulfide bonds. The PEG end groups were decorated with folate (FA) to obtain active targeting, while DOX was trapped into the micelles during their self-assembly. These micelles were specifically internalized into tumour cells through folate receptor-mediated endocytosis, the disulfide bonds were immediately cleaved in response to the high level of glutathione, and in turn DOX was released. An in vivo antitumor effect was evaluated in BALB/c mice bearing 4T1 tumour model, where these DOX@star-PECLss-FA micelles showed the best efficacy when compared with those of free DOX, untargeted micelles, and redox-insensitive micelles.

### 4.4. Other Hyperbranched Copolymers

Poly(2-hydroxyethyl methacrylate) (PHEMA) was used as macromolecular initiator to generate poly(l-lactide)-poly(ethylene glycol) (PLLA-PEG) as side chains, thus obtaining a brush-shaped amphiphilic block copolymer, which was further conjugated with a monoclonal antibody against CD105 (i.e., TRC105), a macrocyclic chelator for ^64^Cu-labeling, and finally loaded with doxorubicin to generate multifunctional theranostic unimolecular micelles [92]. The DOX-loaded micelles showed a pH-dependent drug release profile and a uniform size distribution. Noninvasive PET imaging were carried out in 4T1 murine breast tumour-bearing mice, and a higher level of tumour accumulation was demonstrated for ^64^Cu-labeled targeted micelles when compared with the nontargeted ones.

Paclitaxel was nanoencapsulated into unimolecular micelles based on hydrophobically derivatized hyperbranched polyglycerols (dHPGs), for use as mucoadhesive intravesical agents against non-muscle-invasive bladder cancer [58]. Two different types of dHPGs (HPG-C10-polyethylene glycol (PEG) and polyethyleneimine (PEI)-C18-HPG) were synthesized to obtain nanoparticles with hydrodynamic radii of <10 nm, and incorporation of paclitaxel did not affect their size. When compared with the PEI-C18-HPG formulation, HPG-C10-PEGs showed the best drug release profile and it was also the only stable formulation in acidic urine. In vivo, nude mice with orthotopic KU7-luc tumors were intravesically instilled with phosphate buffered saline, Taxol, or paclitaxel/HPG-C10-PEG. The mucoadhesive HPG-C10-PEG formulation of paclitaxel was significantly more effective in reducing orthotopic tumour growth than Taxol and was well tolerated.

A summary of drug-loaded unimolecular micelles, which were tested in vivo, are summarized in Table 1.

## 5. Conclusions

Properly designed amphiphilic copolymers, once dispersed in aqueous solution, can form unimolecular micelles, i.e., single molecular architectures characterized by a hydrophobic core and a stabilizing hydrophilic shell. Compared to conventional micelles, which are thermodynamic aggregates of amphiphiles above their CMC, unimolecular micelles are stable to many environmental changes, such as dilution and temperature. These are important advantages with regards to their potential applications as drug delivery systems administered in vivo. In this review, recent advances on complex polymeric structures, which are able to generate unimolecular micelles as a drug delivery system, has been discussed. Generally, complex and hindered polymeric structures, characterized by highly branched and strongly hydrophilic chains, yield unimolecular micelles with enhanced stability. A strong emphasis has been put on analytical techniques, such as DLS, TEM, and CMC determination, which can be used to unambiguously assess the formation of unimolecular micelles, and discriminate them from multimolecular aggregates. In the last section, recent examples of unimolecular micelles, used to encapsulate and release hydrophobic drugs to treat several pathologies, have been presented. Their drug loading and release performances, as well as their activity in both in vitro and in vivo conditions, were analyzed, highlighting how their thermodynamic stability results in important features for biological applications, such as long blood circulation, undesired drug leaching and reduced systemic toxicity.

## Figures and Tables

**Table 1:** Unimolecular micellar systems used for in vivo drug delivery.

Polymer System	Polymer Architecture	Drug Encapsulated	Applications	In Vivo Tests	Reference
H40-PCL-b-P(OEGMA-Gd-FA)	Multi-arm star block copolymer	Paclitaxel	Tumor therapy & MRI contrast agent	MR imaging in rats	[70]
H40-PLA-PEG-OCT	Multi-arm block copolymer	Thailandepsin-A	Neuroendocrine cancer therapy	Antitumor efficacy in mice	[71]
H40-PLA-b-TPGS	Multi-arm block copolymer	Docetaxel	Antitumor effect of drug-loaded nanoparticles	Antitumor activity in mice	[72]
H40-PLA-PEG-Apt	Aptamer-conjugated multi-arm star block copolymer	Doxorubicin	Targeted therapy for prostate cancer	Higher level of DOX found in mice tumor tissue	[73]
H40-P(LG-Hyd-DOX)-b-PEG-OCH_3_/cRGD/NOTA	Multi-arm block copolymer conjugated with cRGD and macrocyclic chelator	Conjugated Doxorubicin	Cancer-targeted drug delivery and positron emission tomography imaging	Higher level of tumor accumulation in mice	[74]
β-CD-(PCL-PAEMA-PPEGMA)_21_	21-arm star-like triblock polymer	Doxorubicin	Tumor therapy & (CT) imaging	Antitumor efficacy in mice	[79]
PAMAM-PLA-PEG-OCH3/Cy5.5/GE11	Multi-arm star block copolymer	Aminoflavone	Triple negative breast cancer therapy	Antitumor efficacy in mice	[84]
PAMAM–PVL–PEG–OCH_3_/Cy5/KE108	Multi-arm star block copolymer	AB3	Medullary thyroid cancer therapy	Anticancer efficacy in mice	[85]
PAMAM–PVL–PEG–OCH_3_/Cy5/KE108	Multi-arm star block copolymer	Thailandepsin-A	Neuroendocrine cancer therapy	Antitumor efficacy in mice	[28]
PAMAM–PVL–PEG–Cy5.5/CTB	Multi-arm block copolymer	Dehydroepiandrosterone (DHEA)	Therapy for loss of retinal ganglion cells (glaucoma)	Inhibitory effects on RGC layer degeneration in mice	[86]
PAMAM–PVL–PEG	Multi-arm block copolymer	Rapamycin	Preventing neointima-caused (re)stenosis after open surgery	Inhibitory effect on intimal hyperplasia in rats	[87]
PAM-PGlub-PEG	Multi-arm block copolymer	1,2-diaminocyclohexane-platinum(II)	Lung cancer therapy	PK & Antitumor efficacy in mice	[88]
PDI-star-(PLA-b-PEEP)_8_	Core star block copolymer	Camptothecin	Fluorescence-guided cancer therapy	Tumor growth-inhibitory effect in mice	[90]
star-PECLss-FA	Redox responsive-four-arm block copolymer	Doxorubicin	Targeted anticancer drug delivery	Antitumor effect in mice	[91]
PHEMA-PLLA-PEG-TRC105	Brush-shaped block copolymer	Doxorubicin	pH-controlled targeted drug delivery and PET	Tumor uptake in mice	[92]
PEI-C18-HPG and HPG-C10-PEG	Derivatized hyperbranched polyglycerols	Paclitaxel	Intravesical bladder cancer therapy	Tumor growth inhibition in mice	[58]
